# The elucidation of the dual role of Beclin-1 in ischemic stroke through systems biology modeling

**DOI:** 10.1016/j.isci.2025.113270

**Published:** 2025-08-07

**Authors:** Jun Seok Cha, Jinyoung Kim, Junyoung Cho, Jungho Lee, Jiyoon Kim, Dongwoo Chae

**Affiliations:** 1 Department of Pharmacology, Yonsei University College of Medicine, Seoul 03722, Republic of Korea; 2 Department of Pharmacology, Graduate School of Medical Science, Brain Korea 21 Project, Yonsei University College of Medicine, Seoul, Republic of Korea; 3 Department of Pharmacology, College of Medicine, The Catholic University of Korea, Seoul 06591, Republic of Korea; 4 Department of Medical Sciences, Graduate School, The Catholic University of Korea, Seoul 06591, Republic of Korea; 5 Department of Pharmacology, School of Medicine, Eulji University, Daejeon 34824, Republic of Korea; 6 Institute for Aging and Metabolic Diseases, College of Medicine, The Catholic University of Korea, Seoul 06591, Republic of Korea

**Keywords:** Cardiovascular medicine, Neuroscience, Cell biology

## Abstract

Beclin-1 plays a pivotal role in the interplay between autophagy and apoptosis in ischemic stroke, influencing both cell survival and death. We developed a mathematical model incorporating the dual role of Beclin-1 to simulate Beclin-1-induced autophagy and apoptosis under varying ischemic stress conditions. The model predicts a critical threshold of Beclin-1 expression, beyond which apoptosis is triggered, with this threshold decreasing as stress severity increases. To validate the model predictions, we conducted *in vitro* Beclin-1 overexpression and knockdown experiments under mild and severe oxygen-glucose deprivation (OGD) conditions and *in vivo* Beclin-1 knockdown in a photothrombotic mice model. The experiments demonstrated that Beclin-1 overexpression increases Caspase activation under severe OGD, while knockdown reduces it; the opposite effects were observed under mild OGD. Simulations suggest that modulating Beclin-1 expression could extend the therapeutic window for thrombolysis. Our approach provides insights into the dual roles of Beclin-1 and highlights potential strategies for neuroprotection.

## Highlights

• A model reveals a critical Beclin-1 expression window for neuroprotection in stroke
• Ischemic stress narrows the protective window and lowers the apoptotic threshold
• Beclin-1 knockdown protects in severe stress; overexpression helps in mild stress
• Modulating Beclin-1 levels may broaden the therapeutic window for stroke treatment

## Graphical abstract

**Figure:**
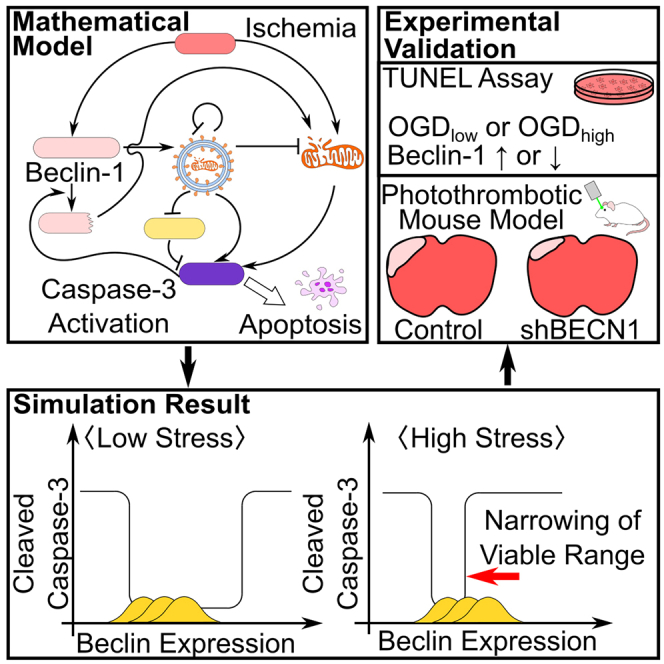

## Introduction

Ischemic stroke is a leading cause of death and disability worldwide. While thrombolysis is an effective treatment, its utility is limited by a narrow therapeutic window, creating an urgent need for neuroprotective strategies that can be applied before reperfusion. Previous studies have shown that both autophagy and apoptosis are simultaneously activated in the ischemic penumbra.^1^^,^^2^

Autophagy, primarily an adaptive response, plays a crucial role in degrading damaged organelles and recycling cellular components. In the context of ischemic stroke, selective autophagy of mitochondria, or mitophagy, is a key cytoprotective mechanism. Beclin-1, a protein essential for autophagy induction, is upregulated in focal ischemia^3^ and enhances mitophagy.^4^ However, excessive Beclin-1 expression has also been reported to promote apoptosis.^5^^,^^6^^,^^7^

Hypoxia stabilizes hypoxia-inducible factor-1α (HIF-1 α) and adenovirus E1B 19-kDa-interacting protein 3 (BNIP3)/BNIP3-like (BNIP3L). This process activates Beclin-1 by promoting its dissociation from Bcl-2.^8^ Concurrently, hypoxia decreases mitochondrial membrane potential (Δφ) and triggers mitochondrial inner membrane permeability transition (MPT),^9^ resulting in the release of pro-apoptotic factors such as cytochrome *c*.^9^ Additionally, BNIP3 and FUN14 domain-containing 1 (FUNDC1) act as mitophagy receptors,^10^ and BNIP3-mediated Beclin-1 activation exerts a neuroprotective effect by enhancing mitophagy. However, Caspase-mediated cleavage of Beclin-1 generates a C-terminal fragment that promotes mitochondrial outer membrane permeabilization (MOMP).^11^ Furthermore, excessive Beclin-1-induced autophagy can be harmful due to the accumulation of autophagosomes^12^ or the selective autophagy of anti-apoptotic proteins.^13^

In this study, we adopt a system-level approach that integrates mathematical modeling with laboratory experiments to elucidate the dual role of Beclin-1 in apoptosis. Our investigation focuses on three key questions: 1) What is the quantitative relationship between Beclin-1 expression and apoptosis? 2) What factors influence this relationship? 3) What therapeutic strategies could be developed to enhance neuroprotection in ischemic stroke?

## Results

### Mathematical model of beclin-1-induced autophagy and apoptosis

**Figure 1:**
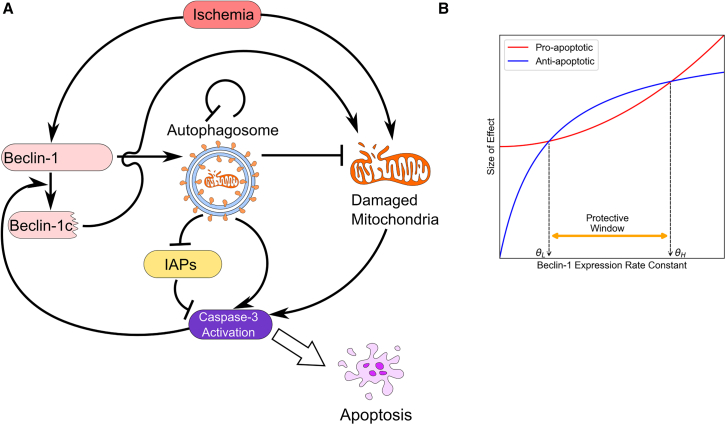
Mathematical model of the dual role of Beclin-1 (A) A schematic diagram of the interaction network of Beclin-1 induced autophagy and apoptosis. Ischemic stress concomitantly upregulates Beclin-1 and promotes mitochondrial damage. Beclin-1 induces the formation of autophagosomes and promotes the removal of damaged mitochondria via mitophagy. Pro-apoptotic mediators released from damaged mitochondria promote Caspase activation. Excessive accumulation of autophagosomes can directly activate Caspase or indirectly reduce the apoptotic threshold through the autophagic degradation of IAPs. Active Caspase-3 cleaves Beclin-1 to produce Beclin-1C that causes mitochondrial damage. Autophagic flux is saturated at high levels of autophagosomes. (B) Beclin-1 upregulation leads to increases in both anti-apoptotic and pro-apoptotic effects, the net effect of which is determined by their relative magnitudes. The model predicts the existence of two critical thresholds, $\theta_{L}$ and $\theta_{H}$, representing the minimum and maximum levels of Beclin-1 compatible with cellular survival.

We developed a mathematical model to capture the dynamics of Beclin-1-induced autophagy and apoptosis, based on a simplified interaction network (Figure 1A). The model employs a system of ordinary differential equations (ODEs) (see STAR Methods for details) to describe the behavior of key molecular players: Beclin-1 (B), cleaved Beclin-1 ($B_{c}$), autophagosomes (A), damaged mitochondria (M), and active Caspase-3 (C), under varying levels of ischemic stress (S). Although lysosomes and inhibitor of apoptosis proteins (IAPs) are not explicitly included in the equations, their roles in the saturable elimination of autophagosomes and active Caspase-3 are incorporated through a Michaelis-Menten formalism.

Our model suggests that the cytoprotective effects of Beclin-1 primarily arise from its role in facilitating the autophagic clearance of damaged mitochondria. As ischemic stress (S) increases, mitochondrial damage escalates accordingly. In response, autophagic flux (J) is upregulated due to increased Beclin-1 expression, mitigating the stress. However, autophagic flux eventually reaches a plateau, constrained by the limited availability of lysosomes.

Conversely, Beclin-1 promotes apoptosis through three distinct mechanisms.

(1) Mechanism 1: Caspase-3 (and Calpain)-mediated cleavage of Beclin-1 generates a fragment, Beclin-1C, which promotes mitochondrial outer membrane permeabilization (MOMP).^11^
(2) Mechanism 2: The accumulation of autophagosomes upon Beclin-1 activation serves as a platform for Caspase-8 activation.^12^^,^^14^
(3) Mechanism 3: The autophagic degradation of IAPs reduces the threshold for apoptosis activation, making cells more susceptible to apoptotic signaling.^13^^,^^15^

The net cellular outcome in response to varying levels of Beclin-1 expression is governed by the balance between its anti-apoptotic and pro-apoptotic effects (Figure 1B). In the viable range where the anti-apoptotic effect exceeds the pro-apoptotic effect of Beclin-1, the cell can survive. Outside the viable range, the cell will be driven to apoptosis.

### Model simulations reproduce the dual effect of Beclin-1 expression

To evaluate the impact of the anti- and pro-apoptotic mechanisms included in our model, we conducted a series of simulations over a 48-h period, with ischemic stress applied from 0 to 24 h. Given the inherent uncertainty in the relative expression levels and half-lives of key molecules and organelles, we employed Latin hypercube sampling to generate 10,000 parameter sets that span a wide range of plausible biological scenarios (see STAR Methods for details). The parameter ranges used for sampling are detailed in Table 1.

**Table 1:** Ranges of parameter values used for Latin hypercube sampling and the representative parameter values

Parameter	Description	Range	Representative value
S	Ischemic stress level	0–10	1
$\alpha_{B}$	Baseline rate of Beclin-1 expression	0.1–5	1
$\beta_{B}$	Rate of Caspase-mediated cleavage of Beclin-1	0.5–2	1
$\gamma_{B}$	Rate of Beclin-1 inactivation	0.5–2	1
$\gamma_{B_{C}}$	Rate of Beclin-1C inactivation	0.5–2	1
$\alpha_{A}$	Formation rate of autophagosome	0.5–2	1
$\beta_{A}$	Rate of autophagic flux	0.5–2	1
$\varphi_{A}$	Saturation threshold of autophagic flux	5–20	10
$\alpha_{M}$	Stress induced formation rate of dysfunctional mitochondria	0.5–2	1
$\sigma_{M}$	Beclin1c induced formation rate of dysfunctional mitochondria	25–100	50
$\gamma_{M}$	Rate of mitophagy	0.5–2	1
$\alpha_{C}$	Rate of Caspase activation due to mitochondrial damage	0.0025–0.01	0.005
$\sigma_{C}$	Rate of Caspase activation due to autophagosomes	0.0005–0.002	0.001
$\mu_{C}$	Autocatalytic rate constant of Caspase	1 fixed	1
$\gamma_{C}$	Degradation rate of Caspase	0.3 fixed	0.3
$\varphi_{C}$	Caspase level at which formation/degradation rate is half maximal	0.15 fixed	0.15
$\sigma_{J}$	Autophagic degradation efficiency of IAPs	0.005–0.02	0.01
$u_{B}$	Sensitivity of Beclin-1 expression to stress	0.5–2	1
$u_{M}$	Sensitivity of mitochondrial damage to stress	0.5–2	1

**Figure 2:**
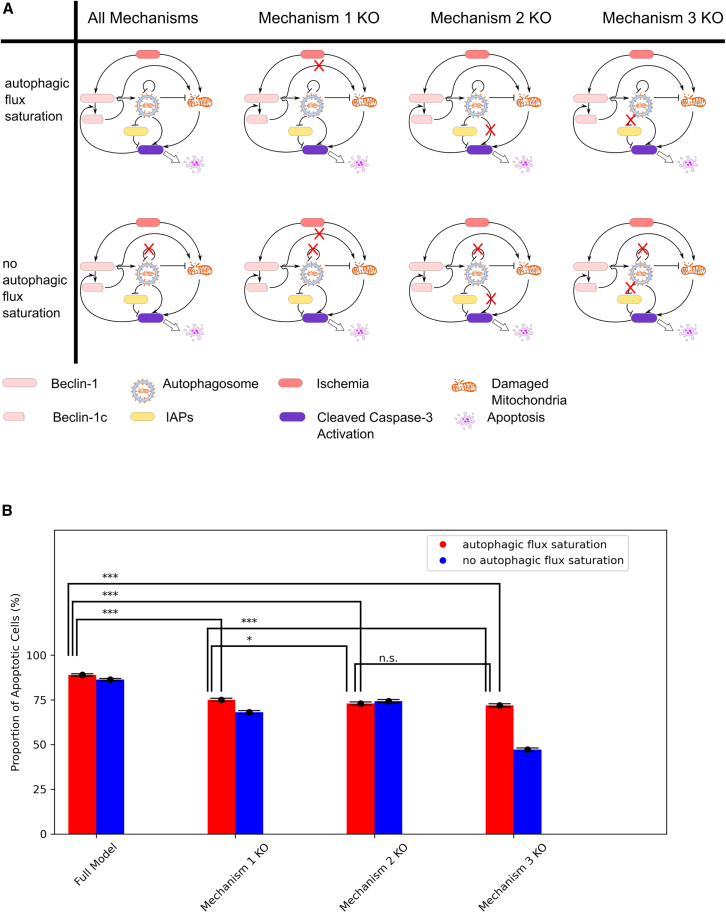
The impact of Beclin-1 expression on apoptosis across different scenarios (A) A schematic representation of the 8 simulation scenarios used to evaluate the effects of Beclin-1 expression on apoptosis. Scenarios are divided into two main groups: one with normal autophagic flux and another with an elevated saturation threshold ($\varphi_{A}$ = $10^{6}$), ensuring no autophagic flux saturation. Each group is further subdivided based on the elimination of one of the three key pro-apoptotic mechanisms. (B) The fraction of apoptotic cells observed across the 8 scenarios (simulations for *n* = 10,000 sampled parameter combinations). Inhibition of any of the three pro-apoptotic pathways significantly reduced the percentage of apoptotic cells. Notably, in the absence of autophagic flux saturation, the survival advantage was markedly enhanced. The error bars are the 95% confidence interval for the proportion estimated using the asymptotic normal approximation. All possible comparisons between the groups were conducted using the chi-square test and Bonferroni’s correction was applied to the *p*-values. ∗∗∗: *p* < 0.001, ∗: *p* < 0.05, n.s.: not significant.

We structured our analysis into eight scenarios (Figure 2A), determined by whether autophagic flux was saturated or not, and whether all three pro-apoptotic mechanisms of Beclin-1 were intact or if one was knocked out. The scenario with autophagic flux saturation and all pro-apoptotic mechanisms intact was labeled the wild-type scenario. Each scenario was assigned 10,000 parameter variants, which were identical to the wild-type scenario except for modifications to autophagic flux saturation or the removal of specific pro-apoptotic mechanisms. Autophagic flux saturation was prevented by increasing the saturation threshold $\varphi_{A}$ to $10^{6}$. The three pro-apoptotic mechanisms were knocked out as follows:

(1) Mechanism 1: Caspase-mediated cleavage of Beclin-1 was disabled by setting the cleavage rate ($\beta_{B}$) to zero.
(2) Mechanism 2: Autophagosome-mediated activation of Caspase was knocked out by setting the activation rate ($\sigma_{C}$) to zero.
(3) Mechanism 3: Autophagic degradation of inhibitor of apoptosis proteins (IAPs) was inhibited by setting the degradation efficiency ($\sigma_{J}$) to zero.

Our simulation results revealed that inhibiting any of the three pro-apoptotic mechanisms reduced the overall rate of apoptosis. Specifically, eliminating Mechanism 1, 2, or 3 led to a decrease in apoptosis from 89.0% in the wild-type to 75.1% (*p* < 0.001), 72.9% (*p* < 0.001), and 71.9% (*p* < 0.001), respectively. Eliminating mechanism 2 or 3 had a slightly but significantly larger impact on apoptosis compared to eliminating mechanism 1 (*p* = 0.02 and *p* < 0.001, respectively). There was no significant difference between the impact of eliminating mechanism 2 or 3 on apoptosis (*p* = 1.00). Increasing the autophagic flux saturation threshold alone had a modest protective effect, reducing apoptosis to 86.3% (*p* < 0.001). However, when this increase in saturation threshold was combined with the inhibition of Mechanism 1, 2, or 3, the reduction in apoptosis was more pronounced, with rates falling to 68.1% (*p* < 0.001), 74.3% (*p* < 0.001), and 47.2% (*p* < 0.001), respectively.

These findings underscore the significant contribution of all three pro-apoptotic mechanisms to apoptosis induction. Moreover, our model predicts that the saturation of autophagic flux amplifies the pro-apoptotic effects of Beclin-1.

### Higher levels of ischemic stress lower the apoptotic threshold of Beclin-1 expression

To explore the relationship between ischemic stress levels and Beclin-1 expression in regulating apoptosis, we analyzed the predicted 48-h Caspase-3 levels across 10,000 parameter variants, stratified by both stress intensity and Beclin-1 expression rate. For each combination, we calculated the fraction of variants that exhibited apoptosis at 48 h, defined by a Caspase-3 level greater than 0.5.

**Figure 3:**
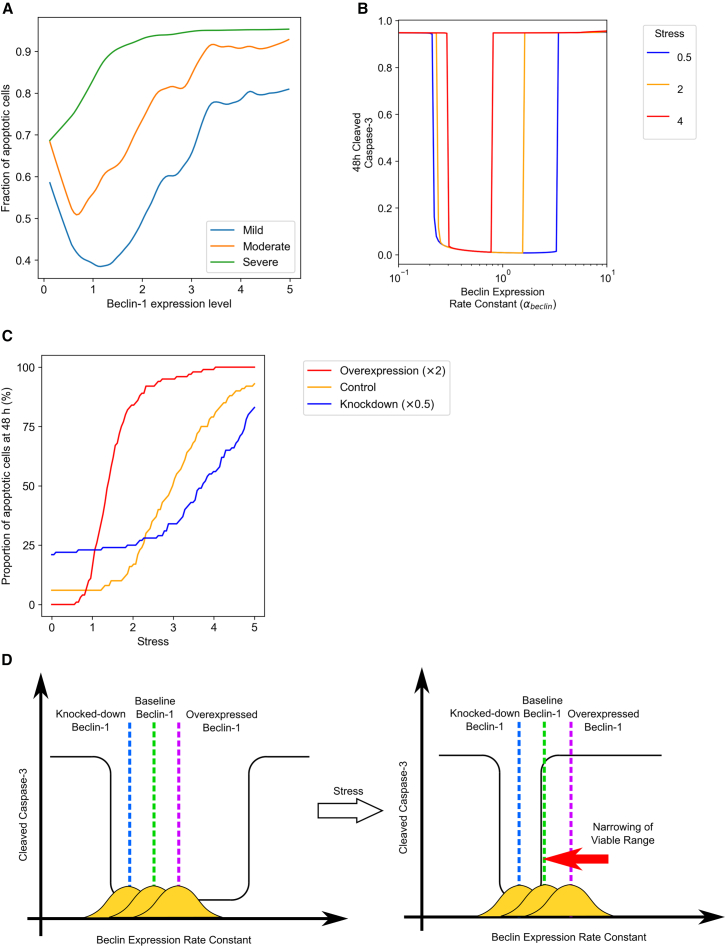
The interplay between apoptosis, Beclin-1 expression, and stress severity (A) The relationship between apoptosis and Beclin-1 expression levels under varying stress conditions: mild (0 < S ≤ 1), moderate (1 < S ≤ 2.5), and severe (2.5 < S ≤ 5). Under mild and moderate stress, an optimal level of Beclin-1 expression minimizes apoptosis. However, under severe stress, increasing Beclin-1 expression consistently leads to a higher probability of apoptosis. (B) The effect of ischemic stress severity on the anti- and pro-apoptotic thresholds of Beclin-1 expression. A simulation grid was employed, varying Beclin-1 expression rates ($\alpha_{B}$) from 0.1 to 10. The results highlight how stress severity shifts the balance between survival and apoptosis, narrowing the window of optimal Beclin-1 expression. See also Figure S1. (C) Simulated outcomes demonstrating that Beclin-1 upregulation and downregulation have opposing effects on apoptosis depending on the level of stress. Under mild stress, Beclin-1 upregulation reduces apoptosis, while under severe stress, downregulation of Beclin-1 becomes beneficial for cell survival. See also Figure S2. (D) Schematic illustrating how Beclin-1 is cytoprotective at low stress levels but promotes apoptosis at high stress levels. As stress increases, the viable range of Beclin-1 expression narrows, with the upper limit of the survival window approaching the baseline Beclin-1 level.

Figure 3A illustrates that under mild (0 < S < 1) and moderate (1 < S < 2.5) ischemic stress, there exists an optimal level of Beclin-1 expression associated with the lowest probability of apoptosis. Initially, increasing Beclin-1 expression exerts a protective effect; however, as expression levels surpass this optimal threshold, Beclin-1 begins to exert a cytotoxic influence. In contrast, under severe stress conditions (S > 2.5), any increase in Beclin-1 expression consistently elevates apoptotic rates.

To gain a deeper understanding of these results, we selected a representative parameter set, referred to as the R variant, with values specified in Table 1. We then simulated 48-h Caspase-3 levels under varying stress intensities (S = 0.5, 2, and 4) and Beclin-1 expression levels ranging from 0.1 to 10. The results (Figure 3B) confirmed the existence of a viable “window” of Beclin-1 expression levels that are compatible with cellular survival, supporting our initial hypothesis. Notably, increasing stress levels were predicted to narrow this survival window by lowering the upper threshold of Beclin-1 expression. Further sensitivity analysis (Figure S1) revealed that most parameter perturbations primarily influenced the pro-apoptotic threshold (the upper limit of the survival window), with the rate of Caspase-mediated cleavage of Beclin-1 ($\beta_{B}$) and the efficiency of autophagosome formation ($\alpha_{A}$) being a critical factor affecting both the lower and upper thresholds.

To generate experimentally testable hypotheses, we conducted *in silico* experiments in which Beclin-1 expression was either doubled or halved, incorporating cell-to-cell variability by adding random noise sampled from a standard normal distribution with a standard deviation of 0.5 to the basal expression levels (with negative values set to zero). These simulations, using the R variant as a reference, suggested that under mild ischemic stress, Beclin-1 upregulation inhibits apoptosis, while downregulation promotes it. Conversely, under severe ischemic stress, downregulating Beclin-1 expression proved beneficial, while upregulation increased cytotoxicity (Figure 3C). The protective effect of Beclin-1 at low stress can be explained by its basal expression being close to the lower limit of the survival window. Conversely, the cytotoxic effect at high stress arises from the narrowing of the survival window, with the upper limit approaching the baseline Beclin-1 level (Figure 3D).

Two extreme scenarios were further examined: (i) When autophagosome formation is highly inefficient ($\alpha_{A}$ is reduced to half of the R variant), even moderate stress resulted in 100% apoptosis. In this case, higher Beclin-1 expression significantly reduced the likelihood of apoptosis (Figure S2A). (ii) When autophagic flux is saturated ($\varphi_{A}$ is reduced 10-fold of the R variant), simulations indicated that lower Beclin-1 expression promoted cellular survival (Figure S2B).

### Oxygen-glucose deprivation increases Beclin-1 cleavage and Caspase-3 activation

**Figure 4:**
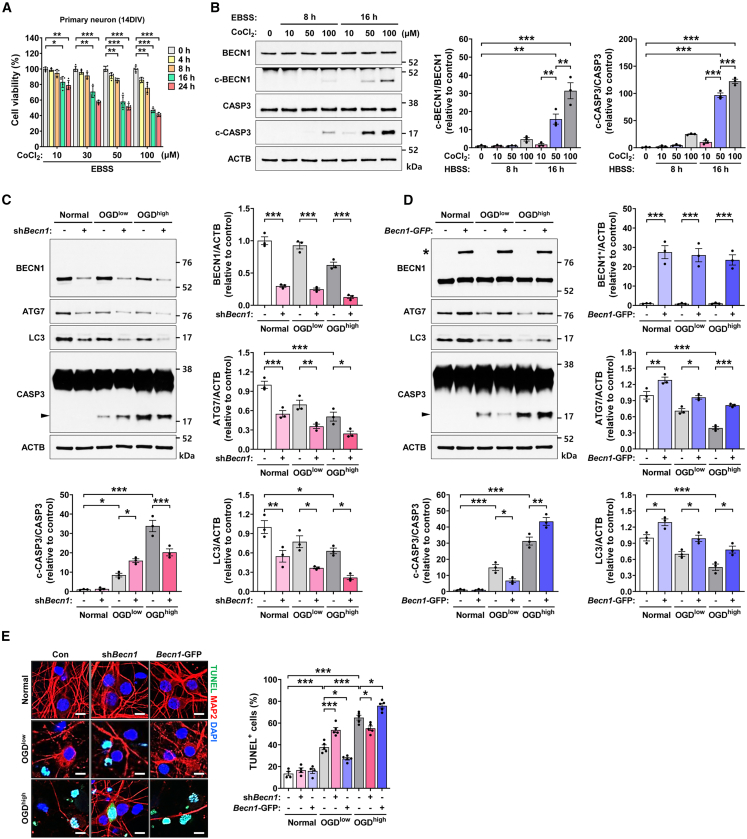
Experimental validation of the dual role of Beclin-1 in primary cortical neurons under oxygen-glucose deprivation (A) The cell viability assay (*n* = 5) demonstrates that increased severity of cobalt chloride (CoCl_2_)-induced hypoxic stress leads to a dose- and time-dependent decline in cell viability. (B) Immunoblot analysis (*n* = 3) reveals that hypoxic stress enhances cleavage of both Beclin-1 and Caspase-3 in a dose- and time-dependent manner. (C) Immunoblot analysis (*n* = 3) demonstrates that Beclin-1 knockdown decreases autophagy, indicated by levels of ATG7 and LC3, independent of OGD conditions. However, Beclin-1 knockdown had OGD level dependent contrasting effects on cleaved Caspase-3 levels following Beclin-1 knockdown in primary neurons under low OGD (10 μM CoCl_2_ for 16 h) and high OGD (50 μM CoCl_2_ for 16 h) conditions. (D) Immunoblot analysis (*n* = 3) illustrates that Beclin-1 overexpression increases autophagy, indicated by the levels of ATG7 and LC3, independent of OGD conditions. However, Beclin-1 overexpression had OGD level dependent contrasting effects on cleaved Caspase-3 in primary neurons under low and high OGD conditions. (E) TUNEL staining (*n* = 4 for control, *n* = 5 for low OGD and high OGD) shows that under low OGD conditions, Beclin-1 knockdown increases cell death whereas Beclin-1 overexpression decreases it; under high OGD conditions, these effects are reversed. (Scale bar, 10 μm) All data are presented as mean ± SEM. ∗: *p* < 0.05, ∗∗: *p* < 0.01 and ∗∗∗: *p* < 0.001 by one-way ANOVA with Tukey’s test.

We performed validation experiments to support the model predictions. To simulate hypoxic conditions *in vitro*, mouse primary cortical neurons were treated with increasing concentrations of cobalt chloride (CoCl_2_) and subjected to glucose deprivation using Earle’s Balanced Salt Solution (EBSS). We observed that higher doses of CoCl_2_ reduced cell viability in a dose- and time-dependent manner (Figure 4A). Western blot analysis revealed that CoCl_2_ concentration correlated with increased cleavage of Beclin-1 and activation of Caspase-3 after 8 and 16 h of treatment, both occurring in a dose-dependent manner (Figure 4B). These findings indicate that ischemic stress induces both Beclin-1 cleavage and apoptosis.

### Effects of Beclin-1 knockdown and overexpression on neuronal cell fate

To further investigate the role of Beclin-1 in neuronal cell fate, we examined the effects of Beclin-1 knockdown and overexpression under mild (10 μM CoCl_2_ for 16 h) and severe (50 μM CoCl_2_ for 16 h) oxygen-glucose deprivation (OGD) conditions. Beclin-1 knockdown was achieved using Beclin-1-specific shRNA plasmid, while Beclin-1 overexpression was induced by transfecting cells with a GFP-tagged Becn1 ORF clone. Regardless of OGD conditions, Beclin-1 knockdown led to decreased autophagy, indicated by biomarkers ATG7 and LC3, while Beclin-1 overexpression led to increased autophagy (Figures 4C and 4D). However, whether Beclin-1 levels positively or negatively correlated with Caspase-3 activation was dependent on OGD conditions. Under mild OGD conditions, Beclin-1 knockdown led to an increase in Caspase-3 activation, whereas under severe OGD conditions, it resulted in decreased Caspase-3 activation (Figure 4C). In contrast, Beclin-1 overexpression decreased Caspase-3 activation under mild OGD but increased it under severe OGD (Figure 4D). TUNEL assay results further supported these findings, showing that Beclin-1 knockdown was cytotoxic under mild OGD but cytoprotective under high OGD, while Beclin-1 overexpression was cytoprotective under mild OGD but cytotoxic under severe OGD. (Figure 1E). These results successfully support our model-based hypothesis that Beclin-1 plays a dual role depending on the severity of ischemic stress: higher Beclin-1 expression is protective under mild ischemic conditions but becomes cytotoxic under severe ischemic stress.

### Effect of Beclin-1 knockdown in a mouse model of ischemic stroke

**Figure 5:**
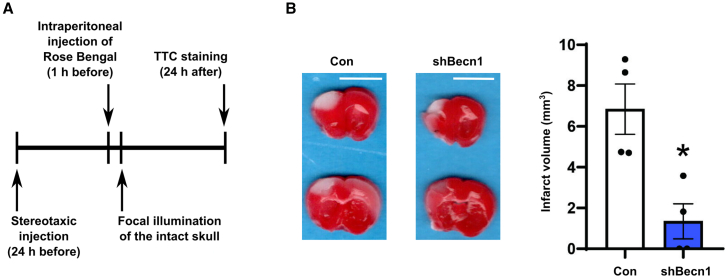
Experimental validation of the effect of Beclin-1 knockdown in the Rose Bengal photothrombotic ischemic mouse model (A) Schematic timeline of the experimental procedure. (B) TTC staining results (*n* = 4) show that Beclin-1 knockdown reduced the infarct volume induced by Rose Bengal photothrombosis. Scale bar, 5 mm. All data are presented as mean ± SEM. ∗*p* < 0.05 by Student’s t test. ∗: *p* < 0.05.

To investigate the role of Beclin-1 in ischemic stroke pathology *in vivo*, we used a photothrombotic ischemic mouse model. Lentiviral vectors carrying either Beclin-1 shRNA or a control sequence were injected into the peri-infarct region, followed by the induction of photothrombotic ischemic stroke using Rose Bengal one day later (Figure 5A).

Quantification of infarct volumes by TTC staining revealed a statistically significant reduction in infarct size in the Beclin-1 shRNA group compared to the control vector group (Figure 5B). These results confirm that the protective effect of Beclin-1 knockdown under conditions of severe ischemic stress, as predicted by our computational modeling, is consistent *in vivo*.

### Simulation suggests interventions that prolong the “golden hour” of thrombolysis

**Figure 6:**
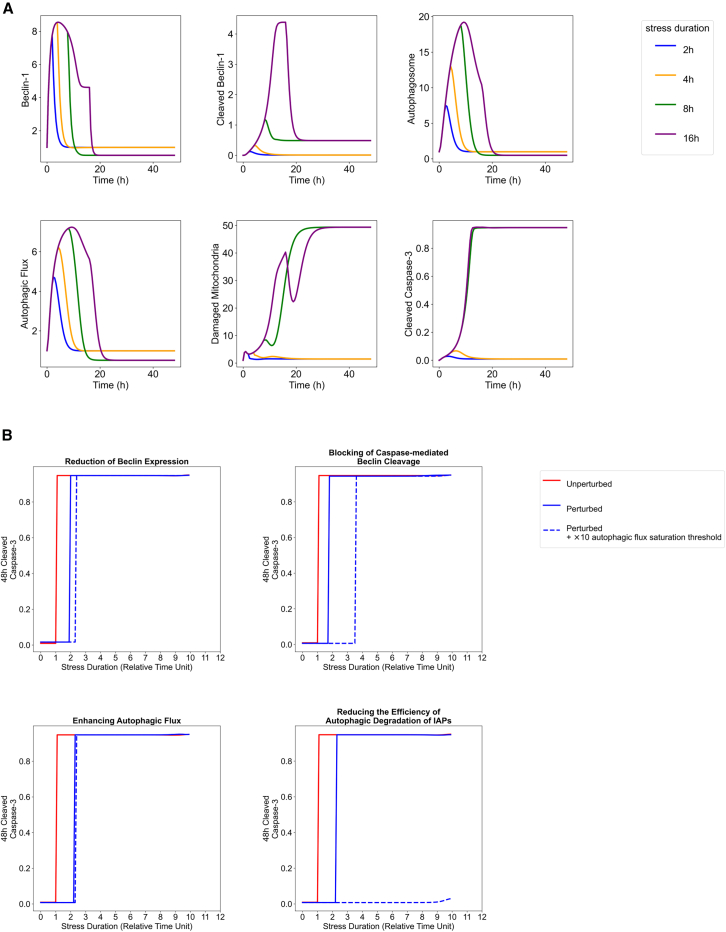
Hypothetical simulations of varying ischemic durations (A) Severe ischemic stress (S = 5) leads to abolished autophagy and apoptosis. The viable range of Beclin-1 expression was set to be the range where Caspase-3 expression is under 0.5. (B) Interventions prolong the “golden hour”. Interventions tried are the reduction of Beclin-1 expression level to 50% its default value ($\alpha_{B}$ = 0.5), eliminating Caspase-mediated Beclin-1 cleavage ($\beta_{B}$ = 0), enhancing autophagic flux 5-fold ($\beta_{A}$ = 5), reducing the efficiency of the autophagic degradation of IAPs 10-fold ($\sigma_{J}=$ 0.001). For each intervention, we additionally tried increasing the autophagic flux saturation threshold by 10-fold (dashed lines).

We calibrated the stress level to replicate the “golden hour” phenomenon in thrombolysis for ischemic stroke, which states that thrombolysis should be conducted within 4 h to be effective.^16^ The stress level was set to S = 8, ensuring that ischemic stress lasting 4 h or less caused only a transient increase in damaged mitochondria and active Caspase-3 levels, while stress beyond 4 h pushed the system into an apoptotic state (Figure 6A). Specifically, the threshold time for apoptosis was 4.8 h for S = 8 (Figure 6B).

To explore potential therapeutic strategies, we conducted a local perturbation analysis of parameters that could serve as drug targets. In these simulations, the 4.8-h threshold was set as the relative time unit for stress duration. First, we evaluated the impact of reducing Beclin-1 expression by 50%. As anticipated, this intervention successfully extended the golden hour by 1.9-fold (Figure 5B). Additionally, we explored the effects of modulating the three key pro-apoptotic mechanisms associated with Beclin-1. Eliminating Beclin-1 cleavage by Caspase (Mechanism 1), abolishing autophagosome-mediated Caspase activation (Mechanism 2), and reducing the autophagic degradation of IAPs by 10-fold (Mechanism 3) extended the golden hour to 1.7, 2.2, and 2.2-fold, respectively (Figure 5B). The benefits of reducing Beclin-1 expression or applying the three strategies when combined with a 10-fold higher autophagic flux saturation threshold were greater, with reducing Beclin-1 expression and knocking out Mechanisms 1, 2, and 3 further extending the golden hours by 2.3, 3.5, 2.3, and 9.9-fold, respectively.

In summary, our simulations suggest that inhibiting Beclin-1 expression is a potential therapeutic strategy to extend the golden hour. Inhibiting the three pro-apoptotic mechanisms in our model is expected to achieve similar effects, and concomitantly raising the saturation threshold of autophagic flux would further enhance the therapeutic benefit.

## Discussion

This study explores the dual role of Beclin-1 in regulating autophagy and apoptosis during ischemic stroke, suggesting how its expression level and the severity of ischemic stress influence cell fate. Through a combination of mathematical modeling and *in vitro* experiments, we demonstrate that Beclin-1 can either protect cells from death or promote apoptosis depending on the specific cellular context, particularly the intensity and duration of ischemic stress. Under mild ischemic conditions, Beclin-1 upregulation enhances mitophagy, clearing damaged mitochondria and supporting cell survival. However, as ischemic stress intensifies, the protective capacity of autophagy reaches a limit, beyond which Beclin-1 overexpression shifts from being beneficial to being harmful. This shift is characterized by the activation of Caspase-3 and the induction of apoptosis, as Beclin-1’s role in promoting mitochondrial outer membrane permeabilization and autophagosome accumulation outweighs its cytoprotective functions. Mathematical modeling revealed that there exist two critical thresholds of Beclin-1 expression, below and above which apoptosis is triggered. The window of Beclin-1 expression spanned by the two thresholds constitutes the window of Beclin-1 expression level compatible with cellular survival.

Simulations of our model showed that higher ischemic stress narrows this protective window, with the upper apoptotic threshold exhibiting a particularly sensitive response to changes in stress levels. This suggests an experimentally testable hypothesis that under severe ischemic conditions, even moderate overexpression of Beclin-1 can drive cells toward apoptosis. Conversely, reducing Beclin-1 expression or enhancing autophagic flux could expand the window of cyto-protection, potentially delaying the onset of apoptosis and extending the therapeutic window for interventions such as thrombolysis. This model-based hypothesis was subsequently verified through *in vitro* experiments using primary neurons. Under mild and severe OGD conditions, shRNA-mediated knockdown was associated with opposing outcomes of increased and decreased Caspase-3 activation, respectively. Conversely, Beclin-1 overexpression under mild and severe OGD caused decreased and increased Caspase-3 activation. Furthermore, we showed that in the photothrombotic ischemic stroke mouse model - corresponding to the severe OGD conditions *in vitro*, shRNA-mediated knockdown of Beclin-1 reduced infarct size.

The context-dependent effects of Beclin-1 are widely supported in the literature. In various pathological conditions, Beclin-1 has demonstrated contradictory effects across different studies. For instance, in several cancers, Beclin-1 has been shown to suppress tumor growth in some malignancies, such as lung cancer, synovial sarcoma, and laryngeal squamous carcinoma,^17^^,^^18^^,^^19^ while promoting tumor progression in others, such as colon cancer and ER-positive breast cancer.^20^^,^^21^ Similar discrepancies have been observed in studies of cardiomyopathy. One study reported that reduced the proteasomal degradation of Beclin-1 by HSPB6 exerted a cardioprotective effect,^22^ whereas another study found that the Beclin1 knockdown in cardiomyocytes exposed to high glucose in a model of type 2 diabetes-induced cardiomyopathy decreased apoptotic cell death.^23^ Comparable findings have been noted in neuronal ischemia-related pathologies. For example, in SH-SY5Y cells subjected to oxygen-glucose deprivation/reperfusion injury, Beclin-1 knockdown via shRNA transduction attenuated hypoxic preconditioning-induced increases in cell viability.^24^ In contrast, in a different study, Beclin-1 knockdown reduced cell death in kainate- and hypoxia-treated primary neuron cultures.^25^

Numerous studies have employed systems biological approaches to investigate the interactions between autophagy and apoptosis, many reporting crosstalk between these two mechanisms. The models used in these studies differ in the extent to which they incorporate molecular mediators involved in autophagy and apoptosis, as well as in their assumptions regarding the mediators that play a pivotal role in regulating this crosstalk. One study employed a model with 21 variables to capture stress intensity and the concentrations of key mediators.^26^ In contrast, another study developed a more comprehensive model with 94 components, incorporating both autophagic and apoptotic factors, and identified that the cytoplasmic calcium level acts as a “rheostat” that regulates responses between these pathways.^27^ Another study used a minimal model, abstracting various factors, and demonstrated that ER stress can trigger apoptosis at a specific activation threshold, with the temporal dynamics of autophagy and apoptosis inducers being highly sensitive to mTOR activity.^28^ In our study, we incorporated molecular mediators more directly linked to the pathology of neuronal hypoxia, exploring specific pathological contexts, such as the viable window for Beclin-1 levels in relation to stress intensity and the critical period for modulating Beclin-1 expression—the “golden hour”—for potential therapeutic intervention.

The concept of the “golden hour” in thrombolysis^16^ - where timely intervention can significantly reduce ischemic stroke-related damage - gains further support from our simulation of ischemic stress duration. We found that there is a critical period beyond which ischemic stress leads to irreversible apoptotic cell death, even if the stressor is removed. Importantly, our simulations suggest that the strategic inhibition of Beclin-1 expression, the prevention of its cleavage by Caspase, and the enhancement of autophagic flux can significantly extend this golden period, offering new avenues for neuroprotective therapies. For example, drugs that compete with Caspase-3 for binding to the cleavage sites of Beclin-1 (e.g., ABHD5) might be valid therapeutic strategies.^29^ To enhance autophagic flux and thereby reduce autophagosome accumulation, the use of lysosome-enhancing compounds which promote transcription factor EB-mediated lysosome biogenesis could be considered.^30^ Although inhibiting the autophagic degradation of IAPs was most effective in inhibiting apoptosis in our simulations, how this might be achieved pharmacologically is not clear. Overall, our study highlights the potential risks associated with therapeutic strategies that broadly enhance autophagy without accounting for the context-dependent effects of Beclin-1. While promoting autophagy might be beneficial under certain conditions, it could also inadvertently exacerbate cell death under high-stress scenarios, where Beclin-1-mediated apoptosis becomes dominant.

In conclusion, our integrated experimental and modeling approach provides a deeper understanding of Beclin-1’s dual role in ischemic stroke, emphasizing the need for context-specific therapeutic strategies. By identifying critical thresholds and mechanisms through which Beclin-1 modulates cell fate, this work offers valuable insights into the development of targeted interventions aimed at enhancing neuroprotection in ischemic stroke. Future research should focus on exploring the therapeutic potential of modulating Beclin-1 and related pathways in clinical settings.

### Limitations of the study

Several limitations of our study should be acknowledged. Firstly, our mathematical model may not fully capture the complexity of ischemic stroke *in vivo*. In ischemic stroke pathology, ischemic stress presents spatially continuous variations in intensity based on the anatomical relationship to the occluded blood vessels, and the brain’s cellular composition is highly heterogeneous. While we accounted for this diversity using LHS, we were unable to capture the full complexity of the cellular environment, which includes various cell types (e.g., neurons, reactive astrocyte subtypes, and microglia) coexisting. Thrombolytic therapy complications and reperfusion injury, which could introduce more complexities, were also not fully incorporated in this study. Secondly, our focus on Beclin-1, while providing significant insights, may not account for the broader spectrum of molecular pathways involved in ischemic stroke, such as inflammation and oxidative stress. Furthermore, the simulations exploring the impact of ischemic stress duration rely on assumed parameters and simplified scenarios and translating these findings into clinical practice presents additional challenges. For simplification, our simulations only used cell apoptosis as the endpoint for successful ischemia treatment, but other factors such as the risk of hemorrhagic transformations are known to be crucial in ischemia therapy. Future research should incorporate these factors in the model.^31^ For exploring potential drug targets, our model could be extended to incorporate upstream regulators such as mTORC1, AMPK, and ULK1. The implementation of binding and dissociation reactions among BNIP3, Beclin-1, Bax, and Bcl-2 would further render our model closer to reality. Various regulators of autophagy and apoptosis, such as p53, calcium, inositol, DAPK, JNK, and others, could be assimilated into our model framework.

## Resource availability

### Lead contact

Further information and requests for resources and reagents should be directed to and will be fulfilled by the lead contact, Dongwoo Chae (dongy@yuhs.ac).

### Materials availability

Reagents generated in this study are available from the lead contact.

### Data and code availability

• Original western blot images have been deposited at Mendeley data at https://doi.org/10.17632/kd728s6m5k.1 and are publicly available as of the date of publication. Microscopy data reported in this article will be shared by the lead contact upon request.
• All original code has been deposited at Mendeley data at https://doi.org/10.17632/kd728s6m5k.1 and is publicly available as of the date of publication.
• Any additional information required to reanalyze the data reported in this article is available from the lead contact upon request.

## Acknowledgments

This study was supported by the 10.13039/501100003725National Research Foundation of Korea (NRF) funded by the 10.13039/501100014188Ministry of Science and ICT, Republic of Korea (RS-2024-00405790). This study was also supported by the 10.13039/501100003725National Research Foundation of Korea (NRF) funded by the Ministry of Education, Republic of Korea (RS-2022-NR075794). This study was also funded by Brain Korea 21 FOUR Project for Medical Science, 10.13039/501100008005Yonsei University College of Medicine, Seoul, Republic of Korea.

## Author contributions

Conceptualization: J.L, Jiyoon K., D.C.; methodology: Jun Seok C., Jinyoung K., Junyoung C., Jiyoon K., D.C.; software: Jun Seok C., D.C. ; validation: Jinyoung K., Junyoung C. ; formal analysis: Jun Seok C., Jinyoung K., Junyoung C., D.C.; investigation: Jinyoung K., Junyoung C.; writing – original draft: Jun Seok C., Jinyoung K., Junyoung C.; writing – review and editing: J.L., Jiyoon K., DC.; supervision: J.L., Jiyoon K., D.C.; project administration: J.L., Jiyoon K., D.C.; funding acquisition: J.L, Jiyoon K., D.C..

## Declaration of interests

The authors declare no competing interests.

## Declaration of generative AI and AI-assisted technologies **in the writing process**

During the preparation of this work, the authors used ChatGPT to check for grammatical errors and improve the academic tone of the article. After using this tool or service, the authors reviewed and edited the content as needed and takes full responsibility for the content of the publication.

## STAR★Methods

### Key resources table

**Table:** 

REAGENT or RESOURCE	SOURCE	IDENTIFIER
**Antibodies**		

BECN1 antibody	BD Sciences	Cat#612112; RRID:AB_399483
CASP3 antibody	Cell Signaling Technology	Cat#9662; RRID:AB_331439
ATG7 antibody	Cell Signaling Technology	Cat#8558; RRID:AB_10831194
LC3 A/B antibody	Cell Signaling Technology	Cat#4108; RRID:AB_2137703
β-actin (ACTB) antibody	Abcam	Cat#ab8227; RRID:AB_2305186
MAP2 antibody	Santa Cruz Biotechnology	Cat#sc-32791; RRID:AB_627948
Alexa Fluor^TM^ 568-*anti*-mouse IgG	Life Technologies	Cat#A10037; RRID:AB_11180865
Goat anti-Mouse IgG (H + L) Secondary Antibody, HRP	Thermo Fisher Scientific	Cat#31430; RRID:AB_228307
Goat anti-Rabbit IgG (H + L) Secondary Antibody, HRP	Thermo Fisher Scientific	Cat#31460; RRID:AB_228341

**Chemicals, peptides, and recombinant proteins**		

Hank’s Balanced Salt Solution (HBSS)	Thermo Fisher Scientific/Gibco	Cat#14185-052
Papain	Worthington Biochemical	Cat#LK003178
DNAse I	Sigma-Aldrich	Cat#D5025
Neurobasal™ Plus Medium	Thermo Fisher Scientific/Gibco	Cat#A35829-01
GlutaMAX™-1	Thermo Fisher Scientific/Gibco	Cat#35050-061
B-27™ Supplement, serum free	Thermo Fisher Scientific/Gibco	Cat#17504001
Penicillin-Streptomycin	Thermo Fisher Scientific/Gibco	Cat#15140122
Fetal bovine serum (FBS)	Thermo Fisher Scientific/Gibco	Cat#16000-044
Earle’s Balanced Salts Solution (EBSS)	Sigma-Aldrich	Cat#E2888
Cobalt chloride (CoCl_2_)	Sigma-Aldrich	Ca#C8661
Tris-HCl (pH8.0)	Biosesang	Cat#T2016–8.0
EDTA (pH8.0)	Biosesang	Cat#E2002
NaCl	Sigma-Aldrich	Cat#S5886
Glycerol	Sigma-Aldrich	Cat#G5516
Triton X-100	Sigma-Aldrich	Cat#T8787
cOmplete™ Protease Inhibitor Cocktail	Sigma-Aldrich/Merck/Roche	11 697 498 001
TBS with Tween 20 (TBST)	Biosesang	Cat#TR2007-100-74
Skim Milk Powder	MBcell	Cat#MB-S1667
Rose Bengal	Sigma-Aldrich	Cat#198250-25G
Isoflurane	Hana Pharm	Insurance code: 657801261
2,3,5-triphenyltetrazolium chloride	Sigma-Aldrich	Cat#T8877-25G
Paraformaldehyde	Merck/EMD Millipore	8.18715.1000

**Critical commercial assays**		

Cell Counting Kit-8 (CCK-8)	Dojindo Molecular Technologies	Cat#CK04-01
ECL Prime Western Blotting Detection Reagent	Amersham	Cat#RPN2232
P3 Primary Cell 4D-Nucleofector^TM^ X Kit	Lonza Bioscience	Cat#V4XP-3024
4D-Nucleofector® X Unit	Lonza Bioscience	Cat#AAF-1003X
*In Situ* Cell Death Detection Kit, Fluorescein	Sigma-Aldrich/Merck/Roche	Cat#11684795910

**Deposited data**		

Data S1 (Cell viability assay raw data for Figure 4A)	This paper	Deposited in Mendely Data https://doi.org/10.17632/kd728s6m5k.1
Data S2 (Uncropped gel image for Figures 4B–4D)	This paper	Deposited in Mendely Data https://doi.org/10.17632/kd728s6m5k.1
Data S3 (TUNEL assay raw data for Figure 4E)	This paper	Deposited in Mendely Data https://doi.org/10.17632/kd728s6m5k.1
Data S4 (Photothrombotic ischemic mouse model data for Figure 5)	This paper	Deposited in Mendely Data https://doi.org/10.17632/kd728s6m5k.1

**Experimental models: Organisms/strains**		

E14.5 embryos of ICR mice (Hsd:ICR(CD-1®))	KOATECH	N/A
C57BL/6J mice	Orient Bio	N/A

**Recombinant DNA**		

*Becn1* shRNA plasmid	Origene	Cat#TL503112; RRID:N/A
GFP-tagged *Becn1* ORF clone	Origene	Cat#MR207162L4; RRID:N/A
Control shRNA	Yonsei Genome Center, Seoul, Korea	Cat#SHC001
BECN1-specific shRNA	Yonsei Genome Center, Seoul, Korea	Cat#TRCN0000033549

**Software and algorithms**		

Data S5 (python script used for analysis)	This paper	Deposited in Mendely Data https://doi.org/10.17632/kd728s6m5k.1
GraphPad Prism v8	GraphPad	RRID:SCR_002798
ImageJ	Schneider et al.^32^	RRID:SCR_003070
Python v3.11.5	Python Software Foundation	RRID:SCR_008394
NumPy v1.25.2	Harris et al.^33^	RRID:SCR_008633
Pandas v2.1.0	McKinney et al.^34^	RRID:SCR_018214
SciPy v1.11.2	Virtanen et al.^35^	RRID:SCR_008058
SymPy v1.12	Meurer et al.^36^	RRID:SCR_018417
Statsmodels v0.14.0	Seabold et al.^37^	RRID:SCR_016074
MatPlotLib v3.7.2	Hunter^38^	RRID:SCR_008624

### Experimental model and study participant details

#### Primary cortical neuron cultures

Primary cortical neurons were isolated from E14.5 embryos of pregnant ICR mice purchased from KOATECH (Pyeongtaek-si, Gyeonggi-do, Republic of Korea). Embryonic brains were dissected in ice-cold Hank’s Balanced Salt Solution (HBSS; Gibco, 14185-052) to collect cortices. The cortices were washed with fresh HBSS and digested by adding papain (20 U/mL; Worthington, LK003176) and DNase I (1 μg/mL; Sigma-Aldrich, D5025), followed by incubation at 37°C for 10 min. After digestion, the tissues were washed three times with HBSS containing DNase I and carefully triturated with a pipette tip in pre-warmed Neurobasal medium (Gibco, A35829-01) containing 2% B-27 supplement (Gibco, 17504001), 1% GlutaMAX (Gibco, 35050-061), and 1% Penicillin-streptomycin (Gibco, 15140122), supplemented with 5% Fetal bovine serum. Isolated neuronal cells were counted using an Automated Cell Counter (Thermo Fisher Scientific, Waltham, MA, U.S.A) and seeded into a poly-D-lysine (Gibco, A3890401)-coated 6-well plate (7.0 × 105 cells/well). Primary neurons were maintained at 37 °C with 5% CO2 until 14 days *in vitro* (DIV). The the identitiy of the cells were validated via immunofluorescence staining for MAP2, a neuronal marker and were confirmed to be mycoplasma-free using the MycoStripTM Mycoplasma Detection Kit (InvivoGen). All animal procedures were approved by the Institutional Animal Care and Use Committee of the Catholic University of Korea (CUMS-2024-0225-01).

#### Photothrombotic ischemic mouse model

Seven-week-old male C57BL/6J mice (20–25 g) were purchased from Orient Bio (Seongnam-si, Gyeonggi-do, Republic of Korea) and used in this study. The mice were housed in cages under a controlled 12:12 h light:dark cycle at 23°C and were allowed to acclimate to the housing conditions for one week prior to ischemia induction. All experimental procedures were approved by the Animal Care and Use Committee of Eulji University (EUIACUC24-14).

To induce photothrombotic ischemia, Rose Bengal (RB; Sigma, 198250-25G, St. Louis, MO, USA) was dissolved in phosphate-buffered saline (PBS) and administered intraperitoneally at a dose of 5 mg/kg. Green light (560 nm) was then locally applied to the right cerebral cortex (2 mm mediolateral from bregma) using an LED lamp (ZEISS, CL 6000LED) for 6 min to initiate RB aggregation and focal ischemia.

To suppress beclin-1 protein expression, lentiviral particles were stereotactically injected into the peri-infarct region (1.2 mm mediolateral from bregma and −1.9 mm ventral to the skull surface) one day before photothrombosis. Lentiviral particles produced from HEK293T cells that had been transfected with the psPAX2 packing plasmid, pMD2.G envelope plasmid, and pLKO.1 short hairpin RNA (shRNA) plasmid (control shRNA: #SHC001, BECN1-specific shRNA: #TRCN0000033549; Yonsei Genome Center, Seoul, Korea). Anesthesia was induced with 5% isoflurane in oxygen using an induction chamber and maintained at 2% isoflurane. Each mouse was secured in a stereotaxic frame with ear bars. A small burr hole was drilled into the skull, and a Hamilton syringe was used to deliver 200 nL of the lentiviral solution at a flow rate of 50 nL/min.

#### Influence of sex on the results of the study

For the primary neurons, we did not test the sex of the E14.5 embryos used in the experiments. Thus, we cannot evaluate the potential influence of embryonic sex on the results. As only male mice were used for the photothorombotic stroke model, we acknowledge that the potential influence of sex on the experimental outcomes could not be assessed for the *in vivo* experiment.

### Method details

#### Development of the mathematical model

A system of ordinary differential equations (ODE) was used to model the dynamics of the key molecular entities involved in Beclin-1 induced autophagy and apoptosis. The model is organized into three interconnected modules: 1) Beclin-1 turnover and cleavage, 2) Autophagosome biogenesis and clearance, and 3) Mitochondrial damage and Caspase activation. The equations governing these processes are detailed in (Equation 1), (Equation 2), (Equation 3), (Equation 4), (Equation 5), (Equation 6).

(Beclin-1 turnover and cleavage)

(Equation 1) $$\begin{array}{c} \dot{B}=\alpha_{B}\;\cdot \;\left(1+u_{B}\;\cdot \;S\right)-\beta_{B}\;\cdot \;C\;\cdot \;B-\gamma_{B}\;\cdot \;B \end{array}$$

(Equation 2) $$\begin{array}{c} \dot{B_{c}}=\beta_{B}\;\cdot \;C\;\cdot \;B-\gamma_{B_{C}}\;\cdot \;B_{c} \end{array}$$

(Autophagosome biogenesis and clearance)

(Equation 3) $$\begin{array}{c} \dot{A}=\alpha_{A}\;\cdot \;B-J \end{array}$$

(Equation 4) $$\begin{array}{c} J=\frac{\beta_{A}\;\cdot \;A}{1+\frac{A}{\varphi_{A}}} \end{array}$$

(Mitochondria damage and Caspase activation)

(Equation 5) $$\begin{array}{c} \dot{M}=\alpha_{M}\;\cdot \;\left(1+u_{M}\;\cdot \;S\right)+\sigma_{M}\;\cdot \;B_{c}-\gamma_{M}\;\cdot \;J\;\cdot \;M \end{array}$$

(Equation 6) $$\begin{array}{c} \dot{C}=\left(\alpha_{C}\;\cdot \;M+\sigma_{C}\;\cdot \;A+\frac{\mu_{C}\;\cdot \;C}{\varphi_{C}+1-C}\right)\;\cdot \;\left(1-C\right)-\frac{\gamma_{C}\;\cdot \;C}{\varphi_{C}+\sigma_{J}\;\cdot \;J+C} \end{array}$$

(S: Hypoxic stress, B: Beclin-1, $B_{c}$: Cleaved Beclin-1, A: Autophagosome, J: Autophagic flux, M: Damaged mitochondria, C: Caspase).

The model assumes mass-action kinetics for most reactions, except for the clearance of autophagosomes, Caspase-3 activation, and Caspase-3 degradation. Autophagic clearance, constrained by the limited availability of free lysosomes, approaches its maximum rate ($\beta_{A}\cdot \varphi_{A}$) when A ≫ $\varphi_{A}$. Caspase-3 activation is modeled based on the interaction between active and inactive forms, with the total amount of Caspase-3 considered constant over the timescale of interest. The activation rate is limited by the abundance of active Caspase-3, which is typically much lower than the inactive form. Caspase-3 degradation is driven by binding with inhibitor of apoptosis proteins (IAPs), which are assumed to be in excess relative to active Caspase-3. However, since IAPs are also subject to autophagic degradation, increased autophagic flux reduces the rate of Caspase-3 degradation. To capture the saturable nature of these processes, we employed Michaelis-Menten kinetics. A critical feature of the model is that autophagic removal of damaged mitochondria cannot increase indefinitely with higher Beclin-1 expression, reflecting the saturation of cytoprotective effects. Moreover, the saturation of both Caspase-3 formation and degradation leads to zero-order ultra-sensitivity, resulting in a switch-like behavior in Caspase-3 activation and inactivation.^39^

#### Generation of parameter variants using Latin hypercube sampling

Latin Hypercube Sampling (LHS) was used to generate 10,000 parameter variants that comprehensively cover the plausible biological ranges for key model parameters. LHS is a statistical method that ensures efficient sampling of multidimensional parameter spaces by dividing each parameter range into intervals and sampling from these intervals in a stratified manner, minimizing correlation among parameters.

Beclin-1 expression is highly context-dependent, varying according to cell type, stress conditions, and disease states. Under normal physiological conditions, Beclin-1 is constitutively expressed at moderate levels, but its expression can be upregulated in response to autophagic stimuli. Reported half-lives of Beclin-1 range from less than 1 h to 9 h,^40^^,^^41^^,^^42^ depending on the cellular environment and interactions with binding partners.

The rate of autophagosome formation is influenced by multiple factors, including nutrient availability, cellular stress, and the levels of autophagy-related proteins (ATGs). Shah et al. quantified the dynamics of autophagosome turnover, reporting a half-life of approximately 2 h under autophagy-inducing conditions.^43^

The half-life of mitochondria varies with cell type, metabolic activity, and physiological conditions. Mitochondria undergo constant turnover through the processes of mitophagy and biogenesis. Typically, mitochondrial half-lives range from hours to several days, but turnover can be accelerated under stress conditions. For example, in response to high levels of oxidative stress, mitochondrial numbers have been shown to decrease by up to 70% within 3.5 h.^44^

Caspase-3 is a critical executioner Caspase in the apoptotic pathway. Its activation is rapid, but the active form is short-lived, typically having a half-life on the order of minutes to hours due to rapid inhibition by inhibitors of apoptosis proteins (IAPs) or degradation via proteasomal pathways.^45^ Caspase-3 is normally present at low levels in its inactive form and is activated through cleavage in response to apoptotic signals.

Given these biological considerations, we assume that most molecular entities in our model exhibit half-lives on the scale of hours. Under steady-state conditions, their levels are theoretically proportional to the ratio of synthesis to degradation rates. To facilitate mathematical analysis, we used arbitrary units for all molecular entities and assumed that their synthesis and degradation rates were comparable, resulting in steady-state levels of approximately 1. This normalization aids in interpreting the model output without the need for unit conversions.

The range of Caspase-3 levels is constrained between 0 and 1, where a value of 1 indicates full apoptotic activation. Parameters governing the intrinsic dynamics of Caspase-3 activation, including $\mu_{C}$, $\gamma_{C}$, and $\varphi_{C}$, were selected to ensure bistable behavior, a characteristic feature of Caspase activation dynamics. Bistability is crucial for modeling the switch-like behavior between life and death decisions in the apoptotic pathway. To preserve this bistability, these parameters were not subject to LHS, as varying them would disrupt the bistable nature of the system. A detailed mathematical analysis of Caspase-3 activation and deactivation dynamics is provided in the subsequent section.

The parameters governing the impact of mitochondrial damage on Caspase activation ($\alpha_{C}$), autophagosomes on Caspase activation ($\sigma_{C}$), Beclin-1C on mitochondrial damage ($\sigma_{M}$), and autophagic flux on IAP degradation ($\sigma_{J}$) was carefully calibrated to ensure that both cellular survival and apoptosis were possible outcomes. For instance, $\alpha_{C}$ greater than 0.01 consistently resulted in apoptosis, prompting us to set these values below 0.01. This calibration was necessary due to the normalization of molecular entities. Since all pathways converge on either inducing mitochondrial damage or directly activating Caspase-3, these parameters were crucial in achieving realistic and biologically relevant simulation outcomes.

In total, we selected 14 parameters for variation through LHS, focusing on key regulators of autophagy and apoptosis. These parameters include: S, $\alpha_{B}$, $\beta_{B}$, $\gamma_{B}$, $\gamma_{B_{C}}$, $\alpha_{A}$, $\beta_{A}$, $\varphi_{A}$, $\alpha_{M}$, $\sigma_{M}$, $\gamma_{M}$, $\alpha_{C}$, $\sigma_{C}$, $\sigma_{J}$, $u_{B}$, and $u_{M}$ (see Table 1 for full details). Each parameter was assigned a biologically plausible range, based on literature values, estimated half-lives, or known physiological limits. These ranges were defined conservatively to encompass uncertainties in biological data and variations in cell-to-cell responses.

We implemented LHS using the *scipy.stats.qmc.LatinHypercube* function from the *SciPy* Python package, ensuring that all 10,000 parameter sets were evenly distributed across the defined multidimensional parameter space. The steps for generating the parameter variants were as follows.

(1) Parameter normalization: Each parameter range was first normalized to the unit interval [0, 1] to facilitate sampling.
(2) Latin Hypercube Sampling: LHS was then applied to sample 10,000 points from the unit interval for each parameter, ensuring stratified sampling across the entire parameter range. This method guarantees that each parameter range is covered uniformly without clustering, reducing sampling bias and providing a more comprehensive exploration of parameter space.
(3) Mapping to original ranges: The sampled values were subsequently mapped back to the original parameter ranges. For each parameter, the sampled values from the unit interval were scaled to its respective biological range using linear interpolation.
(4) Random seed for reproducibility: To ensure reproducibility of the sampling process, a random seed (1234) was used during the generation of the parameter sets. This allows for exact replication of the 10,000 parameter variants in subsequent analyses.

The 10,000 parameter variants generated using LHS were used to explore the effects of varying Beclin-1 expression and ischemic stress on autophagy and apoptosis. For each variant, simulations were run to model the dynamic changes in Caspase-3 activation and Beclin-1-mediated autophagy under different conditions of ischemic stress, with a focus on identifying key thresholds of apoptotic and cytoprotective behavior.

#### Configuring the parameter values for the Caspase system

Based on the Equation 6, we define activation and inactivation functions for active Caspase-3, $f_{act}\left(C\right)$ and $f_{inact}\left(C\right)$, as follows:

(Equation 7) $$\begin{array}{c} f_{act}\left(C\right)=\left(\alpha_{C}\;\cdot \;M+\sigma_{C}\;\cdot \;A+\frac{\mu_{C}\;\cdot \;C}{\varphi_{C}+1-C}\right)\;\cdot \;\left(1-C\right) \end{array}$$

(Equation 8) $$\begin{array}{c} f_{inact}\left(C\right)=\frac{\gamma_{C}\;\cdot \;C}{\varphi_{C}+\sigma_{J}\;\cdot \;J+C} \end{array}$$

Under physiological conditions, it is plausible to assume that both $\alpha_{C}\cdot M+\sigma_{C}\cdot A$ and $\sigma_{J}\cdot J$ are negligible. Hence, we ignore these external influences on the Caspase system and focus on the intrinsic activation and deactivation dynamics of Caspase-3. Under the assumption of $\alpha_{C}\cdot M+\sigma_{C}\cdot A\approx 0$ and $\sigma_{J}\cdot J\approx 0$, the activation and inactivation functions become:

(Equation 9) $$\begin{array}{c} f_{act}\left(C\right)=\left(\frac{\mu_{C}\;\cdot \;C}{\varphi_{C}+1-C}\right)\;\cdot \;\left(1-C\right) \end{array}$$

(Equation 10) $$\begin{array}{c} f_{inact}\left(C\right)=\frac{\gamma_{C}\;\cdot \;C}{\varphi_{C}+C} \end{array}$$

Then the rate of Caspase activation will be the following:

(Equation 11) $$\begin{array}{c} f_{act}\left(C\right)-f_{inact}\left(C\right)=\frac{\mu_{C}\;\cdot \;C}{\varphi_{C}+1-C}\;\cdot \;\left(1-C\right)-\frac{\gamma_{C}\;\cdot \;C}{\varphi_{C}+C} \end{array}$$

(Equation 12) $$\begin{array}{c} =-\frac{\mu_{C}C}{\left(\varphi_{C}+1-C\right)\left(\varphi_{C}+C\right)}\;\cdot \;\left(C^{2}+\left(\varphi_{C}-1-\frac{\gamma_{C}}{\mu_{C}}\right)\;\cdot \;C+\left(-\varphi_{C}+\frac{\gamma_{C}\;\cdot \;\varphi_{C}}{\mu_{C}}+\frac{\gamma_{C}}{\mu_{C}}\right)\right) \end{array}$$

Let us define $g_{1}$, $g_{2}$ as the following and Δ as the determinant of $g_{2}$.

(Equation 13) $$\begin{array}{c} g_{1}\left(C\right)=-\frac{\mu_{C}\;\cdot \;C}{\left(\varphi_{C}+1-C\right)\left(\varphi_{C}+C\right)} \end{array}$$

(Equation 14) $$\begin{array}{c} g_{2}\left(C\right)=C^{2}+\left(\varphi_{C}-1-\frac{\gamma_{C}}{\mu_{C}}\right)\;\cdot \;C+\left(-\varphi_{C}+\frac{\gamma_{C}\;\cdot \;\varphi_{C}}{\mu_{C}}+\frac{\gamma_{C}}{\mu_{C}}\right) \end{array}$$

(Equation 15) $$\begin{array}{c} \Delta =\frac{1}{2}\left({\left(\varphi_{C}-1-\frac{\gamma_{C}}{\mu_{C}}\right)}^{2}-4\left(-\varphi_{C}+\frac{\gamma_{C}\;\cdot \;\varphi_{C}}{\mu_{C}}+\frac{\gamma_{C}}{\mu_{C}}\right)\right) \end{array}$$

Then the following holds:

(Equation 16) $$\begin{array}{c} f_{act}\left(C\right)-f_{inact}\left(C\right)=g_{1}\left(C\right)\;\cdot \;g_{2}\left(C\right) \end{array}$$

A necessary condition for the system to exhibit bistability is for $f_{act}\left(C\right)$ to intersect $f_{inact}\left(C\right)$ at three points in 0≤C≤1. We already know that $g_{1}\left(0\right)=0$, so we know that 0 is one intersection point. We also know the following holds:

(Equation 17) $$\begin{array}{c} g_{1}\left(C\right)<0\;for\;0<C\leq 1 \end{array}$$

So, the necessary and sufficient condition for intersection at three points in 0≤C≤1 is for $g_{2}$ to have two roots at 0<C≤1. We also know that:

(Equation 18) $$\begin{array}{c} g_{2}\left(1\right)=\frac{\gamma_{C}\;\cdot \;\varphi_{C}}{\mu_{C}}>0 \end{array}$$

Meanwhile, the necessary and sufficient condition for the existence of two roots $\gamma_{1}$ and $\gamma_{2}$ of $g_{2}$ in 0<C≤1 is the following:

(Equation 19) $$\begin{array}{c} \text{COND}1:\left(\Delta >0\;\&\;g_{2}\left(0\right)>0\right) \end{array}$$

On the other hand, if COND1 holds, $f_{act}$ and $f_{inact}$ will have three intersections, one at C=0 and two, $\gamma_{1}$ and $\gamma_{2}$ ($\gamma_{1}<\gamma_{2}$), at 0<C<1. We can show that there exists some δ>0 such that $g_{1}\left(C\right)<0$ and $g_{2}\left(C\right)>0$ for any 0<C<δ. So, $f_{act}\left(C\right)-f_{inact}\left(C\right)<0$ for any 0<C<δ. So, C=0 is a stable critical point. $g_{2}$ changes sign when C crosses each root of $g_{2}$, so $f_{act}\left(C\right)-f_{inact}\left(C\right)=g_{1}\left(C\right)\cdot g_{2}\left(C\right)$ will change sign when C crosses each root of $g_{2}$. So, $C=\gamma_{1}$ is an unstable critical point and $C=\gamma_{2}$ is a stable critical point.

Therefore, we can see that COND1 is a necessary and sufficient condition for the system to be bistable. Also, if it is the case that COND1 is bistable, there will be three critical points, $C=0,\gamma_{1},\gamma_{2}$ where $0<\gamma_{1}<\gamma_{2}<1$. Also, $C=0,\gamma_{2}$ will be stable and $C=\gamma_{1}$ will be unstable. If either of the two conditions for COND1 is not met, the system will not be bistable (Figure S3A).

Among a wide range of possible values of parameters that satisfy COND1, we selected the following parameter combinations: $\mu_{C}$ = 1, $\gamma_{C}$ = 0.3, and $\varphi_{C}$ = 0.15. These parameters were found to adequately confer bistabiity to the Caspase activation dynamics (Figure S3A top left panel).

In the following section, we analyze how mitochondrial damage (M), autophagosome abundance (A), and autophagic flux (J) affect the Caspase-3 dynamics.

#### Bifurcation analysis of the Caspase-3 system

To investigate the effect of M, A, and J on Caspase-3 activation, we simplified the system and applied numerical continuation to draw bifurcation diagrams. The stabilities of the critical points were determined by evaluating the sign of $\frac{d}{dC}\left(f_{act}\left(C\right)-f_{inact}\left(C\right)\right)$ at each critical point.

Increase of M and A drive higher Caspase-3 activation. To probe the effect of these factors using bifurcation analysis, let us assume that M and A do not change with time. Then the activation and inactivation function would be the following:

(Equation 20) $$\begin{array}{c} \alpha_{1}=\alpha_{C}\;\cdot \;M+\sigma_{C}\;\cdot \;A \end{array}$$

(Equation 21) $$\begin{array}{c} f_{act}\left(C\right)=\left(\alpha_{1}+\frac{\mu_{C}\;\cdot \;C}{\varphi_{C}+1-C}\right)\;\cdot \;\left(1-C\right) \end{array}$$

(Equation 22) $$\begin{array}{c} f_{inact}\left(C\right)=\frac{\gamma_{C}\;\cdot \;C}{\varphi_{C}+C} \end{array}$$

At low values of $\alpha_{1}$, the system exhibits two stable steady states: a low-Caspase state (survival) and a high-Caspase state (apoptosis). However, as $\alpha_{1}$ increases, these two stable states coalesce and disappear at a critical point, resulting in a saddle-node bifurcation. Beyond this bifurcation, only the high-Caspase apoptotic state remains stable, forcing the cell into apoptosis (Figure S3B left panel).

J plays a dual role in regulating Caspase-3. While higher J reduces M and indirectly inhibits the activation of Caspase-3, excessive autophagic flux can deplete IAPs and decrease the deactivation rate of Caspase-3. To probe the effect of this mechanism using bifurcation analysis, let us assume that J does not change with time. Then the activation and inactivation functions could be written as:

(Equation 23) $$\begin{array}{c} \alpha_{2}=\sigma_{J}\;\cdot \;J \end{array}$$

(Equation 24) $$\begin{array}{c} f_{act}\left(C\right)=\left(\frac{\mu_{C}\;\cdot \;C}{\varphi_{C}+1-C}\right)\;\cdot \;\left(1-C\right) \end{array}$$

(Equation 25) $$\begin{array}{c} f_{inact}\left(C\right)=\frac{\gamma_{C}\;\cdot \;C}{\varphi_{C}+C+\alpha_{2}} \end{array}$$

The term $\alpha_{2}$ in the denominator of $f_{inact}\left(C\right)$ of Equation 9 diminishes the system’s capacity to deactivate Caspase-3. As J increases, the system approaches a critical point where inactivation is no longer sufficient to maintain the low-Caspase state, resulting in a saddle-node bifurcation and a shift to the apoptotic state (Figure S3B right panel).

The above analysis succinctly reveals the dual effect of Beclin-1 induced autophagy on Caspase-3 level. On the one hand, increased Beclin-1 expression increases autophagic flux (J) and mitophagy rate. The resultant reduction of M then lowers $f_{act}\left(C\right)$ and lifts the apoptotic threshold. On the other hand, the rise of autophagosome abundance (A) due to higher Beclin-1 expression increases $f_{act}\left(C\right)$. If Beclin-1 overexpression results in a greater increase of $\sigma_{C}\cdot A$ than a reduction of $\alpha_{C}\cdot M$, the net effect would be higher $f_{act}\left(C\right)$. To shift the balance in favor of greater reduction of $\alpha_{C}\cdot M$, either Beclin-1C mediated mitochondrial damage should be abolished (Mechanism 1), the efficiency of autophagosome-mediated Caspase-3 activation (i.e., $\sigma_{C}$) reduced (Mechanism 2), or the rate of mitophagy increased. Since the rate of mitophagy is influenced by the efficiency of mitophagy ($\gamma_{M}$) and autophagic flux (J), increasing either of the two would enhance the cytoprotective effect of Beclin-1 upregulation. However, since an increase of J also decreases $f_{inact}\left(C\right)$ via autophagic degradation of IAPs (Mechanism 3), increasing J comes at a cost. Therefore, a better strategy would be to increase the efficiency of mitophagy ($\gamma_{M}$) or reduce that of IAP degradation ($\sigma_{J}$).

In summary, therapeutic strategies aimed at inhibiting Mechanisms 1 and 2 achieve their goal by reducing $f_{act}\left(C\right)$, while those that increase the efficiency of mitophagy while reducing that of IAP degradation (Mechanism 3) do so by increasing $f_{inact}\left(C\right)$. The best strategy would depend on the relative contribution of the three mechanisms to apoptosis induction.

#### Oxygen-glucose deprivation (OGD)

An OGD condition was established in primary cortical neurons to mimic ischemic stroke *in vitro*. To create the OGD condition, the complete neurobasal medium was replaced with Earle′s Balanced Salts Solution (EBSS; Sigma-Aldrich, E2888) to induce glucose deprivation. Additionally, cobalt chloride (CoCl_2_; Sigma-Aldrich, C8661) was added to simulate hypoxic conditions. To investigate the dual role of Beclin-1, primary neurons were incubated under low OGD conditions (10 μM CoCl_2_ in EBSS) or high OGD conditions (50 μM CoCl_2_ in EBSS) for 16 h.

#### Cell viability assay

A cell viability assay was conducted using the Cell counting Kit-8 (CCK-8) reagent (Dojindo Molecular Technologies, CK04-01), following the manufacturer’s instructions. Primary cortical neurons were seeded into a 24-well plate (1.4 × 105 cells/well). After the OGD procedure, CCK-8 reagent was added to each well, and the plate was incubated at 37 °C for 1h. The optical density (OD) values were measured at 450 nm using a microplate reader (BioTek, Winooski, VT, U.S.A.).

#### Becn1 knockdown and overexpression

Primary cortical neurons isolated from mouse embryos were immediately transfected with *Becn1* shRNA plasmid (Origene, TL503112) or *GFP*-tagged *Becn1* ORF clone (Origene, MR207162L4) using the P3 Primary Cell 4D-Nucleofector X Kit (Lonza Bioscience, V4XP-3024) and 4D-Nucleofector X Unit (Lonza Bioscience, Basel, Switzerland), according to the manufacturer’s instructions. Briefly, isolated primary neurons in 100 μL of a Nucleocuvette Vessel were placed in the Nucleofector equipment and electroporated with 2 μg of DNA using the P3 Primary Cell Nucleofector Solution. Subsequently, neurons transfected with *Becn1* shRNA plasmid or *GFP*-tagged *Becn1* plasmid were seeded into an 8-well chamber slide (1.0 × 10^5^ cells/well) for TUNEL staining and a 6-well plate (7.0 × 10^5^ cells/well) for immunoblot analysis.

#### Immunoblot analysis

Whole-cell lysates were prepared for immunoblotting by lysing cells in Lysis buffer containing 20 mM Tris-HCl (pH 7.9), 150 mM NaCl, 2 mM EDTA, 5 mM EGTA, 5% Glycerol, 1% Triton X-100, supplemented with Protease Inhibitor Cocktail (Roche, 11 697 498 001), 1 mM phenylmethanesulfonylfluoride (PMSF), 10 mM NaF, and 1 mM Na_3_VO_4_. The lysates were sonicated for 20 s and centrifuged at 13,000 × *g* for 20 min. The supernatant was collected, and protein concentration was determined using the Bradford assay. Proteins were separated by SDS-PAGE and transferred onto nitrocellulose membranes (Thermo Fisher Scientific, 88018). Membranes were blocked with TBS-T containing 5% skim milk for 60 min and probed with primary antibodies against Beclin-1 (BECN1; BD sciences, 612112, 1:1,000 dilution), Caspase-3 (CASP3; Cell signaling Technology, 9662, 1:1,000 dilution), ATG7 (Cell signaling Technology, 8558, 1:1,000 dilution), LC3 A/B (Cell signaling Technology, 4108, 1:3,000 dilution) or β-actin (ACTB; Abcam, ab8227, 1:5,000 dilution). After incubation with HRP-conjugated secondary antibodies, protein bands were detected using ECL Prime Western Blotting Detection Reagent (Amersham, RPN2232).

#### TUNEL staining

To assess the apoptotic neuronal cell death, TUNEL staining was performed using the *In Situ* Cell Death Detection Kit (Roche, 11 684 795 910), according to the manufacturer’s instructions. Primary cortical neurons were seeded into an 8-well chamber slide (1.0 × 10^5^ cells/well). Following the OGD procedure, the neurons were fixed with pre-chilled 4% paraformaldehyde for 30 min and incubated with the TUNEL reaction mixture at 37 °C for 60 min in the dark. Subsequently, cells were blocked with 5% donkey serum for 60 min to prevent non-specific binding, followed by incubation with a primary antibody against MAP2 (Cell signaling Technology, 4542), a marker for neuron. The cells were then incubated with Alexa Fluor 568-*anti*-mouse IgG (Life Technologies, A10037), followed by DAPI staining. Confocal microscopy was conducted using an LSM800 microscope (Carl Zeiss, Oberkochen, Germany), and the percentage of TUNEL^+^ neurons was evaluated using ImageJ software (NIH).^32^

#### TTC staining

At 24 h post-ischemia induction, the mice were euthanized, and their brains were rapidly removed. The brains were coronally sectioned at 1 mm thickness using a brain matrix. All sections were incubated in 1% 2,3,5-triphenyltetrazolium chloride (TTC; Sigma, T8877-25G, St. Louis, MO, USA) at 37°C for 20 min and then fixed in 4% paraformaldehyde (PFA) for one day. Infarct regions were photographed, and infarct volumes were quantified using ImageJ software.

### Quantification and statistical analysis

For Figure 2B, each of the eight conditions was simulated using 10,000 parameter sets generated via Latin hypercube sampling. For each condition, the proportion of apoptotic cells at 48 h was calculated. Among the four conditions involving autophagic flux saturation, pairwise comparisons of the 48-h apoptotic proportions (six pairwise tests) were conducted using the chi-square test. Resulting *p*-values were adjusted for multiple comparisons using Bonferroni correction. The center values displayed in Figure 2B represent the estimated proportions, and the error bars correspond to the 95% confidence intervals calculated via the asymptotic normal approximation. We used Python 3.11.5 for analyzing and simulating model behavior with the help of the following libraries: NumPy v1.25.2,^33^ pandas v2.1.0,^34^ SciPy v1.11.2,^35^ SymPy v1.12,^36^ and statsmodels v0.14.0.^37^ Chi-square tests were performed using SciPy,^35^ and the 95% confidence intervals for proportions were estimated with statsmodels.^37^ Plotting library MatPlotLib v3.7.2^38^ was used for visualization.

For Figures 4A–4E and 5B, all quantitative data are presented as mean ± standard error of the mean (SEM). Comparisons between two groups were conducted using the two-tailed Student’s t test, while comparisons among more than two groups were performed using one-way analysis of variance (ANOVA) followed by Tukey’s post hoc test. These analyses were carried out using GraphPad Prism version 8 (GraphPad Software, Boston, MA, USA). The number of experimental replicates, with a minimum of three per condition, is detailed in the corresponding figure legends.

*P*-values less than 0.05 were considered statistically significant. Significance levels are denoted as follows: n.s.: not significant, *p* < 0.05 (∗), *p* < 0.01 (∗∗), and *p* < 0.001 (∗∗∗).
