# Supplementary material for: The elucidation of the dual role of Beclin-1 in ischemic stroke through systems biology modeling
Source: iScience. 2025 Aug 7;28(9):113270. doi: 10.1016/j.isci.2025.113270 (PMC12396021; doi:10.1016/j.isci.2025.113270)
Supplement: Document S1. Figures S1–S3 [file mmc1.pdf]

iScience, Volume *28*

Supplemental information

The elucidation of the dual role of Beclin-1 in ischemic stroke through systems biology modeling

Jun Seok Cha, Jinyoung Kim, Junyoung Cho, Jungho Lee, Jiyoon Kim, and Dongwoo Chae

Supplemental Figures

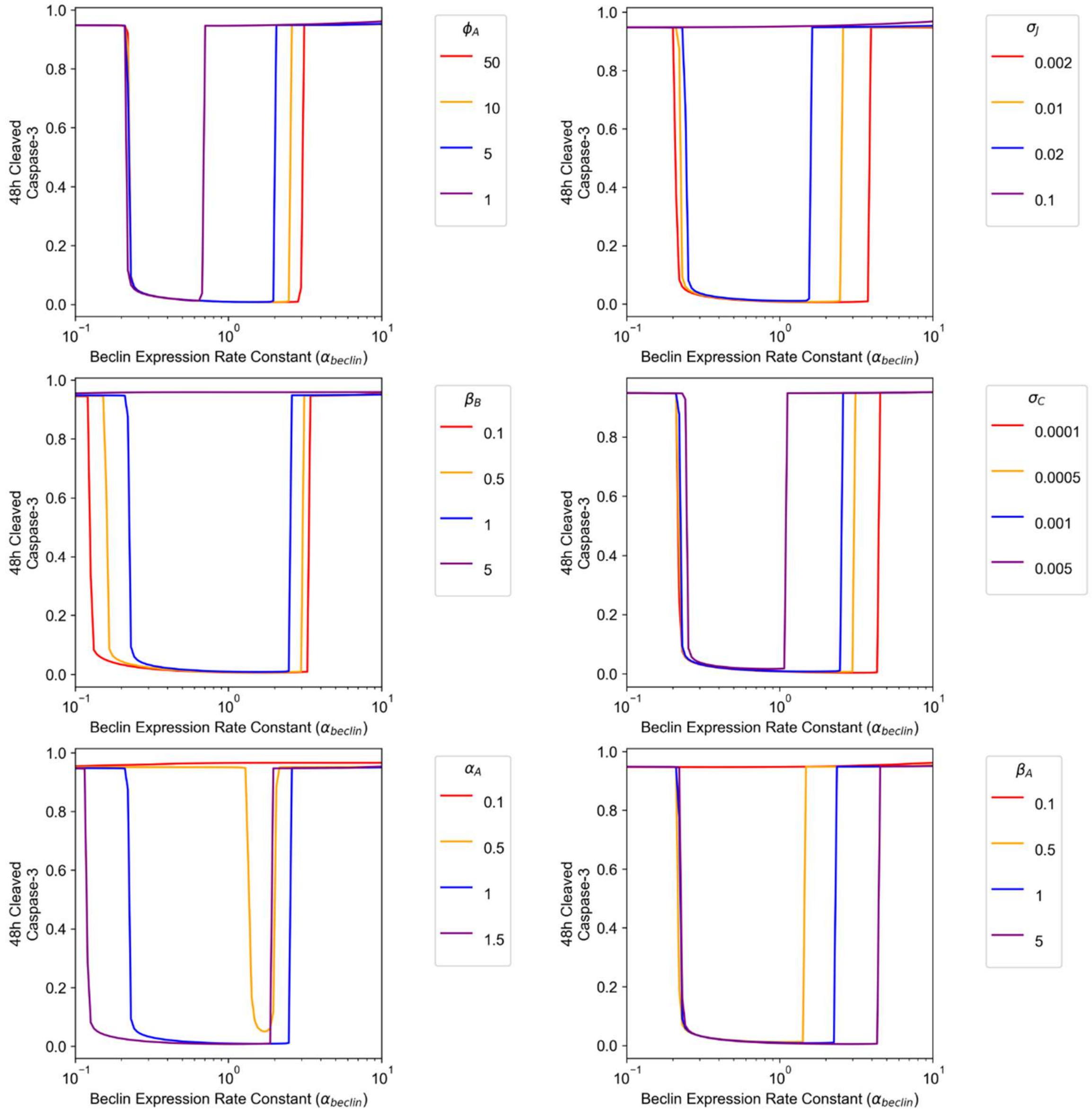

Figure S1. The effect of different parameter perturbations on the Beclin-1 viable window, related to Figure 3B

The effect of difference in parameter values on the anti- and pro-apoptotic thresholds of Beclin-1 expression. Beclin-1 expression rates (α_B) were varied from 0.1 to 10. The parameters examined are saturation threshold of autophagic flux (ϕ_A), autophagic degradation efficiency of IAPs (σ_J), rate of Caspase-mediated cleavage of Beclin-1 (β_B), rate of Caspase activation due to autophagosomes (σ_C), formation rate of autophagosome (α_A), rate of autophagic flux (β_A).

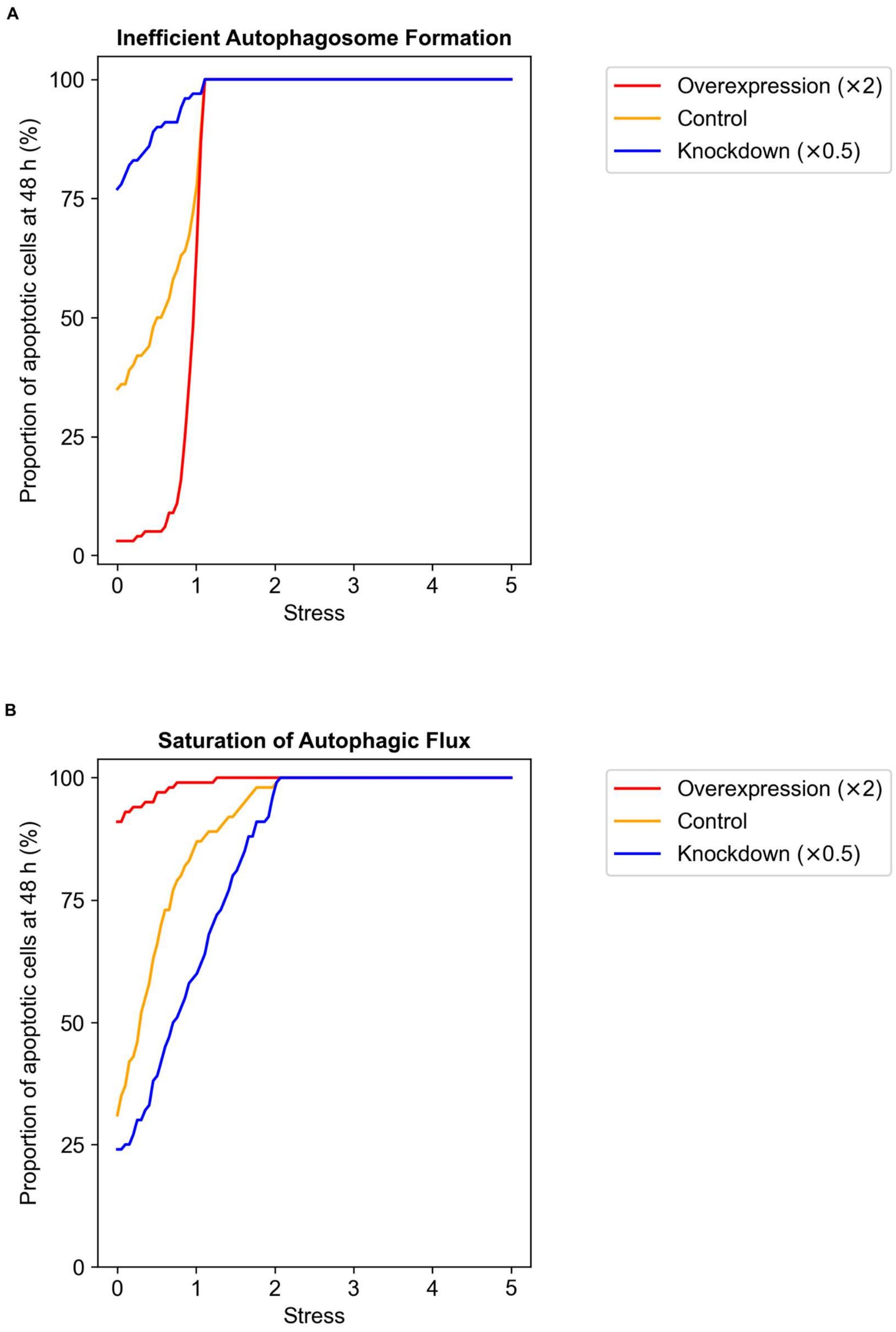

Figure S2. The effect of Beclin-1 modulation in two extreme scenarios, related to Figure 3C
(A) Under inefficient autophagosome formation, Beclin-1 upregulation primarily performs a cytoprotective role. (B) Under autophagic flux saturation, higher Beclin-1 does not contribute to cytoprotective function, and lower Beclin-1 expression increases cellular survival.

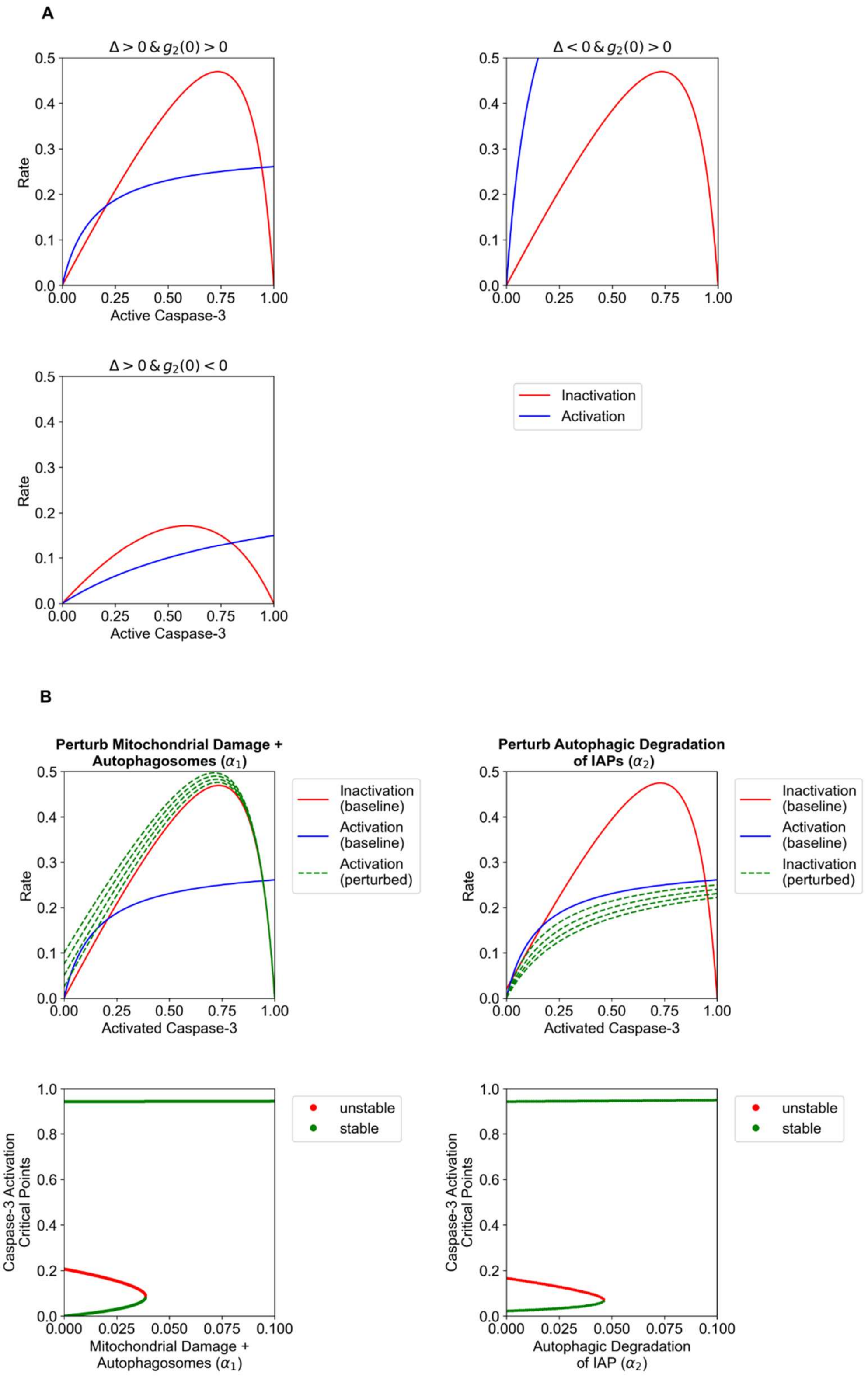

Figure S3. Bistability and saddle node bifurcation of the caspase system, related to STAR Methods bifurcation analysis of the Caspase-3 system

(A) Bistability requires that $\Delta > 0$ and $g_2(0) > 0$. The parameter sets for the top left, top right, and bottom left diagram are $[\mu_C = 1, \varphi_C = 0.15, \gamma_C = 0.3]$, $[\mu_C = 1, \varphi_C = 0.15, \gamma_C = 1.0]$, and $[\mu_C = 1, \varphi_C = 1, \gamma_C = 0.3]$ respectively. The Δ for the top left, top right, and bottom left diagrams are 0.271, -0.289,

and 0.845, respectively. The $g_2(0)$ for the top left, top right, and bottom left diagram are 0.195, 1.0, and -0.400, respectively. (B) Increase of mitochondrial damage, autophagosomes, or autophagic flux cause saddle-node bifurcation beyond critical thresholds, resulting in irreversible induction of apoptosis.
